# Supplementary material for: Live birth in an archosauromorph reptile
Source: Nat Commun. 2017 Feb 14;8:14445. doi: 10.1038/ncomms14445 (PMC5316873; doi:10.1038/ncomms14445)
Supplement: Supplementary Information — Supplementary Figures, Supplementary Tables, Supplementary Notes and Supplementary References. [file ncomms14445-s1.pdf]

Supplementary Note 1. Detailed description of LPV 30280

Skull. In LPV 30280**,** the posterior part of the skull is exposed in ventral view. No bones can be unequivocally identified except for the posteriormost part of the mandible.

Axial skeleton. In LPV 30280, part of the dorsal and caudal region is preserved. The neurocentral suture is still open in the specimen.

Cervical vertebrae. LPV 30280 preserves the anterior 15 cervical vertebrae. The atlas centrum is slightly disarticulated and exposed in ventral view. It is a roughly rectangular element, similar to *Dinocephalosaurus*. The axis centrum is also exposed in ventral view. Compared with the atlas, the axis is much elongated. Both the dorsal and the ventral margin are slightly concave. Both zygapophyses are well developed. The postzygapophyses are detached and shifted posteriorly. The suture between the neural arch and the centrum cannot be discerned. The remaining vertebrae are exposed in right lateral view. The ventral margin of the third vertebra is distinctly concave, as in *Dinocephalosaurus*. The suture between the neural arch and the centrum is clear. The neural arch overlaps the centrum in lateral view. The 8th vertebra is broken and exposes the cross section. Its cross section clearly shows a hollow centrum and a hollow base of paired neural arches, although the lateral compression is severe. The 15th vertebra only preserves the anterior part. The cross section shows a trace of the existence of a hollow centrum after severe lateral compression.

Dorsal vertebrae. In LPV 30280, there are four probably posterior dorsal neural arches articulated with each other. No centrum below is found. This demonstrates that the dorsal neural arches and centra were not fused. The height of the neural spines is subequal to their length.

Caudal vertebrae. There are 12 caudal vertebrae preserved in LPV 30280. Their exact position in the caudal region cannot be ascertained. The anterior three preserve the neural arches associated with ribs. The centra are somehow lost, again demonstrating the open neuro-central sutures. In the posterior nine vertebrae, both neural arches and centra are preserved, associated with the haemal arches. The caudal neural spines are moderately high compared with the cervical and dorsal ones. Transverse processes are well developed, but their width is no more than the length of the corresponding centrum. The anterior three haemal arches are pointed distally. For the middle four haemal spines, the distal width is more than the proximal width. The two posterior neural arches are preserved in antero-posterior view.

Ribs. In LPV 30280, there is no trace of the preservation of atlantal ribs. The axial ribs extend posteriorly beyond the intervertebral articulation between the 5th and 6th cervical vertebrae. The third cervical ribs extend posteriorly at least beyond the intervertebral articulation between the 6th and 7th cervical vertebrae. The fourth cervical ribs extend posteriorly at least beyond the intervertebral articulation between the 7th and 8th cervical vertebrae. The posterior extension of the 5th cervical ribs remains unclear. The 6th cervical ribs extend posteriorly at least to the middle of the 10th cervical vertebra. The 7th cervical ribs extend posteriorly at least beyond the

intervertebral articulation between the 10th and 11th cervical vertebrae. The 8th cervical ribs extend posteriorly at least to the middle of the 12th cervical vertebra. The 9th cervical ribs extend posteriorly at least beyond the intervertebral articulation between the 12th and 13th cervical vertebrae. Posterior extension of left cervical ribs remains unclear because of weathering. Cervical ribs extend posteriorly parallel to the neck axis. They are dichocephalous, with anterior processes.

Dorsal ribs are short and stout compared with the cervical ribs. In LPV 30280, all of the visible dorsal ribs are holocephalous.

There are three caudal ribs preserved, associated with the relevant caudal vertebrae in LPV 30280. Caudal ribs are curved and separated from relevant caudal vertebrae.

Some gastral ribs are also preserved. Each gastral rib is composed of one angulated medial element and two lateral elements.

Hind limb. Part of the hind limbs is preserved in LPV 30280. Only the distal end of one femur is preserved. Nothing else can be said about this bone. Both tibiae are preserved but with only the distal part exposed. The distal end of the tibia is much more delicate than the fibula. The fibula is distinctly curved. Compared with the proximal end, the distal end of the fibula is distinctly expanded.

There are two ossified tarsals preserved in each limb. Both are round elements. The larger one is identified as the astragalus, and the smaller one as the calcaneum.

All metatarsals are preserved. The first metatarsal is the shortest, while the fourth is the longest. The first metatarsal is different from other metatarsals in that its distal end is not expanded. The 5th metatarsal is straight without a hooked posterior margin, and is distinctly longer than the first metatarsal.

The phalangeal formula of the pes is unclear since the preservation is incomplete. However, the 4th digit preserves a complete sequence with six phalanges. This indicates mild hyperphalangy in the pes of LPV 30280, as in IVPP V13898. The length of the first phalanx of the 5th digit is subequal to the metatarsal, but significantly shorter than other metatarsals.

Supplementary Note 2. Character description

If a character is originally from Benton and Allen[1], Jalil[2] or Dilkes[3], it is noted as B/J/D plus the original character sequence in the relevant reference. If the coding for a character is different from Rieppel *et al.*[4], a note is given in the text below. The character sequence has been changed to reflect the anatomical grouping. The character list was prepared using NDE Version 0.5.0.

1. **Dimensions of skull (D1):**
   0. Midline length greater than maximum width
   1. Midline length less than maximum width
2. **Skull low and narrow with short and narrow postorbital region (J38):**
   0. absent
   1. present
3. **Relative length of snout (D2, J64):**

0. less than 50% of total skull length
1. equal or more than 50% of total skull length

4. Premaxilla shape (Modified after D6):

An additional state, i.e., a convex ventral margin, is added to accommodate the shape of the premaxilla of *Dinocephalosaurus.*

0. horizontal ventral margin
1. down-turned ventral margin
2. convex ventral margin

5. Premaxilla, dorsomedial process (B1, J25):

Rhynchosaurus is recoded from 0 to 1 because the dorsomedial process of the premaxilla is essentially absent in *Rhynchosaurus*[5], forming a single external opening in the middle of the rostrum. *Prolacerta* would be coded as 0 based on the reconstruction by Gow[6]. However, Modesto and Sues[7] stated that none of the specimens of *Prolacerta* available preserve the tip of the dorsomedial process of the premaxilla clearly. So this character is recoded from 1 to ?. *Jesairosaurus* is recoded from ? to 0 based on personal observation of MNHN ZAR 06.

0. extends between narial openings
1. reduced

6. Premaxilla and prefrontal (D7):

Boreopricea is recoded from ? to 0 based on Benton and Allen[1]. *Jesairosaurus* is recoded from ? to 0 based on personal observation of MNHN ZAR 06.

0. no contact
1. contact present

7. Premaxilla, posteroventral process (Modified after D8):

Jesairosaurus is recoded from ? to 0 based on personal observation of MNHN ZAR 06.

0. reduced
1. well developed and excluding maxilla from external naris

8. Premaxilla and maxilla, form of suture above dentigerous margin (D17):

Both Modesto and Sues[7] and Gottmann-Quesada and Sander[8] described a supralabial foramen in *Protorosaurus* that extends anteriorly to the suture with the premaxilla, so *Protorosaurus* is recoded from 0 to 1. Benton and Allen[1] described marked "blood vessel/nerve pits on the surface of the bone above the tooth row" in the maxilla of *Boreopricea.* It is not clear if they are homologous with the supralabial foramen in other basal archosauromorphs which extends to the suture line with the premaxilla. So this character is recoded from 0 to ? for *Boreopricea.* The PKU specimen of *Macrocnemus* indeed shows the presence of a supralabial foramen. However, this foramen by no means extends to the suture line with the premaxilla. Hence, *Macrocnemus* is still coded as 0. Personal examination of *Jesairosaurus* specimens shows that preservation in this region is not unequivocal, so *Jesairosaurus* is recoded from 0 to ?. Personal examination of *Langobardisaurus* specimens confirms

the absence of such a notch. Therefore, *Langobardisaurus* is recoded from ? to 0.

0. simple vertical or diagonal contact
1. notch present in maxilla

9. Maxilla (D16):

Boreopricea is recoded from 1 to 0 based on Benton and Allen[1].

0. horizontal ventral margin
1. convex ventral margin

10. External naris (D10):

The snout tip in *Boreopricea* is not preserved[1], so *Boreopricea* is recoded from 0 to ?. *Jesairosaurus* is recoded from ? to 0 based on personal observation of MNHN ZAR 06. *Macrocnemus* is recoded from ? to 0.

0. separate
1. single, medial naris

11. External naris location (D11, J26):

The region of the external naris is not preserved in *Boreopricea*[1], so this character is recoded from 0 to ? for *Boreopricea*. *Langobardisaurus* is recoded from ? to 1 based on personal observation of MFSN 1921. The holotype of *Dinocephalosaurus orientalis* shows that the external naris is much closer to the lateral margin than the middle line of the skull. *Jesairosaurus* is recoded from ? to 1 based on personal observation of MNHN ZAR 06.

0. marginal
1. close to midline

12. External naris shape from lateral view (D12, J26):

Reconstruction by Gregory[9] shows a round external naris for *Trilophosaurus*, so it is recoded from 1 to 0. *Jesairosaurus* is recoded from ? to 1 based on personal observation of MNHN ZAR 06.

0. rounded
1. elongate

13. Nasal groove:

0. absent
1. present

14. Antorbital fenestra (D5):

Megalancosaurus and *Langobardisaurus* are recoded from ? to 0 based on personal observation. *Boreopricea* is recoded from ? to 0 based on Benton and Allen[1].

0. absent
1. present

15. Nasal, shape of cranial margin at midline (D13, J49):

Boreopricea is recoded from 1 to ? since the reconstruction of the anterior region of the nasals is largely imaginative.

0. strongly convex with anterior process
1. transverse with little convexity

16. Nasals, relative length (B2, D18):

The reconstruction by Carroll[10] contradicts with that by Gow[6], so *Youngina* is recoded from 1 to ? temporarily. *Macrocnemus* is recoded from ? to 1 based on the GMPKU specimen. *Boreopricea* is recoded from 0 to ? since the anterior part of the nasal is incompletely preserved.

0. shorter than frontals
1. longer than frontals

17. Frontal, shape of dorsal surface next to sutures with postfrontal and parietal (D20):

0. flat to slightly concave
1. longitudinal depression with deep pits

18. Frontals and parietals, ratio of lengths (D19):

Jesairosaurus is recoded from ? to 0 based on personal observation.

0. more than 1
1. equal or less than 1

19. Parietal table (D26):

Megalancosaurus is recoded from ? to 0 based on CCSR 63115. *Macrocnemus* is recoded from 1 to 1+2 based on the personal observation. *Langobardisaurus* is recoded from ? to 2 based on personal observation of MCSNB 2883. *Boreopricea* is recoded from 0 to ?.

0. broad
1. constricted without sagittal crest
2. sagittal crest present

20. Parietals, median contact in adult (D25):

Megalancosaurus is recoded from ? to 0 based on CCSR 63115. *Macrocnemus* is recoded from 0 to 1 based on the GMPKU specimen. *Boreopricea* is recoded from 0 to1 based on the description of Benton and Allen[1].

0. suture present
1. parietals fused with loss of suture

21. Parietal, shape of median border (D28):

Boreopricea is recoded from 0 to ?.

0. level with skull table
1. drawn downwards to form ventrolateral flange

22. Pineal foramen (B4, D27):

Rhynchosaurus is recoded from 0 to 1 based on Benton[5]. *Macrocnemus* is recoded from 0 to 1.

0. present and relatively large
1. reduced or absent

23. Postparietals (modified from J3, J14, D29):

Megalancosaurus is recoded from ? to 2 based on CCSR 63115.

0. paired
1. fused
2. absent

24. Septomaxilla (D14):

Youngina is recoded from ? to 0. *Prolacerta* is recoded from ? to 0 based on Modesto and Sues[7].

0. present
1. absent

25. Lacrimal (J15):

Macrocnemus, *Tanystropheus*, *Protorosaurus* and *Boreopricea* are recoded from 1 to 0. *Prolacerta* is recoded from ? to 0 based on Modesto and Sues[1]. *Jesairosaurus* is recoded from ? to 0 based on personal observation of MNHN ZAR 06.

0. large
1. small or absent

26. Lacrimal contact with nasal (B5, D15):

The posteroventral suture of the nasal of the holotype of *Dinocephalosaurus orientalis* cannot be delineated unequivocally.

0. contacts nasal and reaches external naris
1. contacts nasal but does not reach naris
2. does not contact nasal or reach naris

27. Lacrimal extent (B6):

Langobardisaurus is recoded from ? to 1 based on personal observation of MFSN 1921. *Jesairosaurus* is recoded from ? to 0 based on personal observation of MNHN ZAR 06.

0. element runs forward from the orbit
1. restricted to the orbital rim in lateral view

28. Prefrontals (D125):

0. separate along midline
1. meet along midline

29. Postfrontal dimensions (B7):

Euparkeria is recoded from 1 to 0 based on Ewer[11]. *Protorosaurus* is recoded from ? to 0 based on Gottmann-Quesada and Sander[8].

0. substantial tripartite element
1. short element lacking clear processes

30. Postfrontal, shape of dorsal surface (D21):

0. flat or slightly concave towards raised orbital rim
1. depression present with deep pits

31. Postfrontal (D24):

Youngina is recoded from 0 to 1 following Modesto and Sues[7]. *Langobardisaurus* is recoded from ? to 1 based on personal observation of MFSN 1921.

0. excluded from upper temporal fenestra
1. entering upper temporal fenestra

32. Postorbital and parietal contact (D22):

Macrocnemus is recoded from 0 to 1. *Dinocephalosaurus* is recoded from ? to 1 based on personal observation of the holotype. *Jesairosaurus* is recoded

from ? to 1 based on personal observation of MNHN ZAR 06 and MNHN ZAR 07. *Youngina* is recoded from 1 to 0 following Modesto and Sues[7].

0. present
1. absent

33. Postorbital, ratio of lengths of anteroventral and posterodorsal processes (D23, B8). :

Youngina is recoded from 0 to 1. Dilkes's own figures[3, 12] show that the posterodorsal process of the postorbital is longer than the anteroventral process. So *Mesosuchus* and *Howesia* are recoded from 0 to 1. *Dinocephalosaurus* is recoded from ? to 0.

0. > 1.0
1. < 1.0

34. Jugal, lateral surface above maxilla (D33):

0. continuous
1. lateral shelf present

35. Jugal, posterior process (B10, J28, J42):

Macrocnemus is recoded from 1 to 0 based on personal observation. *Langobardisaurus* is recoded from ? to 0 based on personal observation. *Protorosaurus* is recoded from ? to 0 based on Gottmann -Quesada and Sander[8]. *Prolacerta* is recoded from 1 to 0 based on Modesto and Sues[7]. *Jesairosaurus* is recoded from ? to 0 based on personal observation of MNHN ZAR 06.

0. well-developed but does not extend to the posterior margin of the lower temporal fenestra
1. absent
2. extends nearly to the posterior margin of the lower temporal fenestra

36. Jugal, posterior process (D32):

Jesairosaurus is recoded from 0 to 1 based on personal observation of MNHN ZAR 06. *Boreopricea* is recoded from 0 to -.

0. robust with height > 50% of length
1. slender with height < 50 % of length

37. Lower temporal fenestra (D4, J13):

Langobardisaurus is recoded from ? to 1 following Modesto and Sues[7]. *Trilophosaurus* is recoded from ? to 2 following Modesto and Sues[7].

0. present and closed ventrally
1. present and open ventrally
2. absent

38. Quadratojugal (D35, B11, B12):

Preservation of this region in the holotype of *Dinocephalosaurus* and in *Jesairosaurus* are not unequivocal, so they are recoded from 2 to ?.

0. present with anterior process
1. present without anterior process
2. absent

39. Squamosal, ventral ramus (B9, J4):

Megalancosaurus is recoded from ? to 1 based on personal observation of MFSN 1769. *Langobardisaurus* is recoded from ? to 0 based on personal observation of MFSN 1921. *Protorosaurus* is recoded from ? to 0 based on Gottmann-Quesada and Sander[8].

0. present and extends below quadrate head
1. reduced and cotyle formed for quadrate head

40. Upper temporal fenestra (D3):

0. oval in outline and not elongated caudally
1. elongated caudally with inner surface of parietal and squamosal facing dorsally

41. Supratemporal (B13, D31):

Macrocnemus is recoded from 0 to 1 based on personal observation. *Tanystropheus* is recoded from 0 to ?. *Protorosaurus* is recoded from ? to 1 based on Gottmann-Quesada and Sander[8]. *Boreopricea* is recoded from 0 to ?.

0. present
1. absent

42. Tabular (J2, D30):

0. present
1. absent

43. Vomer and maxilla contact (D38):

0. absent
1. present

44. Ectopterygoid and maxilla contact (D40):

0. absent
1. present

45. Ectopterygoid and jugal contact (D39):

0. restricted with area of contact approximately equal to or less than contact between ectopterygoid and pterygoid
1. ectopterygoid expanded caudally

46. Ectopterygoid, shape along suture with pterygoid (D42):

0. transversely broad
1. posteroventrally elongate and does not reach lateral corner of transverse flange
2. posteroventrally elongate and reaches corner of transverse flange

47. Suture between ectopterygoid and pterygoid (D142):

0. simple overlap of ectopterygoid and pterygoid
1. complex overlap between ectopterygoid and pterygoid

48. Suborbital fenestra, elements contributing to lateral border (D41):

0. ectopterygoid, palatine and maxilla
1. ectopterygoid and palatine contact to exclude maxilla

49. Pterygoids (D126):

Dinocephalosaurus is recoded from ? to 0.

0. join cranially
1. remain separate

50. Quadrate (D36):

Megalancosaurus is recoded from ? to 1 based on personal observation of MFSN 1769.

0. covered laterally
1. exposed laterally

51. Quadrate, posterior emargination in lateral view (J5, D37):

Tanystropheus is recoded from 0 to 1.

0. absent
1. present

52. Quadrate, prominent lateral conch in posterior view (J16, D37):

Euparkeria is recoded from 0 to 1. *Protorosaurus* is recoded from ? to 1 following Gottmann-Quesada and Sander[8].

0. absent
1. present

53. Stapedial foramen (J6):

0. present
1. absent

54. Parasphenoid-basisphenoid in the side wall of braincase (J68):

0. no
1. yes

55. Occipital condyle position (D51, J66):

Preservation of *Boreopricea* does not allow unequivocal coding of this character. *Dinocephalosaurus* is recoded from ? to 0.

0. even with craniomandibular joint
1. cranial to craniomandibular joint
2. caudal to craniomandibular joint

56. Paroccipital process (D52, J7):

0. ends freely
1. reaches suspensorium

57. Post-temporal fenestra (D53, J65):

To quantitatively define this character, we divide the horizontal diameter of the post-temporal diameter to the maximum width of the skull in posterior view. The ratios for different taxa are as follows: *Petrolacosaurus*: 0.15; *Youngina:* 0.12/0.15/0.16; *Trilophosaurus*: 0.14; *Rhynchosaurus*: 0.07; *Mesosuchus*: 0.07; *Howesia*: 0.1; *Prolacerta*: 0.12; *Proterosuchus*: 0.12; *Euparkeria*: 0.09. So *Tanystropheus* and *Boreopricea* is recoded from 0 to ?, while *Rhynchosaurus* are recoded from 0 to 1.

0. large
1. small or absent

58. Basioccipital:

0. forming the ventral boundary of foramen magnum
1. nearly excluded from foramen magnum

59. Supraoccipital (D54):

Dinocephalosaurus is recoded from ? to 0 based on personal observation of the holotype.

0. plate-like
1. pillar-like

60. Prootic, anterior inferior process (D48):

0. absent
1. present

61. Prootic, lateral surface (D47, J70):

0. continuous and slightly convex
1. crista prootica present

62. Opisthotic, club-shaped ventral ramus (D46):

0. absent
1. present

63. Abducens foramina for nerve VI (D49):

0. in dorsum sella
1. between prootic and dorsum sella

64. Internal carotid foramen (D45):

Youngina is recoded from 0 to 1, while *Howesia* is recoded from ? to 1.

0. in the lateral wall of braincase
1. in the ventral surface of parasphenoid

65. Basipterygoid processes orientation (D43):

Prolacerta is recoded from 0 to 1.

0. anterolateral
1. lateral

66. Basicranial joint (D130):

0. metakinetic
1. fused

67. Laterosphenoid (D50, J69):

0. absent
1. present

68. Jaw occlusion (D65):

0. single-sided overlap
1. flat occlusion
2. blade and groove

69. Lower jaw, depth measured at maximum height of adductor fossa relative to length of jaw from tip to articular (D70):

0. < 25 %
1. > 25 %

70. Jaw smphysis (D71, D72):

0. formed largely or wholly by dentary
1. formed only by splenial

71. Jaw symphysis (D127):

0. small
1. extended caudally

72. Dentary-coronoid-surangular profile (D73):

Langobardisaurus is recoded from ? to 1. *Prolacerta* is recoded from 1 to 0 following Modesto and Sues[7].

0. horizontal to convex
1. concave caudal to coronoid

73. Angular, lateral exposure (J17):

Dinocephalosaurus is recoded from ? to 0.

0. large
1. restricted

74. Retroarticular process (J8, J18, D74):

0. absent/reduced
1. well developed

75. Upturned retroarticular process (D75):

Jesairosaurus is recoded from 0 to 1 based on personal observation of MNHN ZAR 06.

0. absent
1. present

76. Lateral mandibular fenestra (D76):

Langobardisaurus is recoded from ? to 0 based on personal observation of MFSN 1921.

0. absent
1. present

77. Anterior surangular foramen (From Modesto and Sues[7], Character 145):

0. absent
1. present

78. Posterior surangular foramen (From Modesto and Sues[7], Character 146):

0. absent
1. present

79. Crown of marginal teeth (D136):

0. single point
1. tricuspid

80. Tooth rows, relative positions of posterior terminations (B14, J62):

Megalancosaurus is recoded from ? to 1 based on Renesto and Dalla Vecchia[13]. *Langobardisaurus* is recoded from ? to 0. *Protorosaurus* is recoded from ? to 0 based on Gottmann-Quesada and Sander[8].

0. posterior dentary teeth lie level with, or behind, posterior maxillary teeth
1. posterior dentary teeth lie anterior to posterior maxillary teeth

81. Curvature of teeth (J63, D58):

Dinocephalosaurus is recoded from 0 to 1.

0. absent
1. present

82. Teeth, cross-sectional shape (J63, D59):

A new state (antero-posteriorly compressed) is added to accommodate the tooth shape of *Trilophosaurus*. *Jesairosaurus* is recoded from ? to 0, while *Prolacerta* is recoded from 1 to 0.

0. oval
1. laterally compressed
2. antero-posteriorly compressed

83. Tooth implantation (D55):

Dinocephalosaurus is recoded from ? to 1. *Prolacerta* is recoded from 0 to 1 following Modesto and Sues[7].

0. subthecodont
1. ankylothecodont
2. pleurodont

84. Caniniform teeth (D56):

Langobardisaurus is recoded from ? to 1, while *Dinocephalosaurus* is recoded from 1 to 0.

0. present
1. absent

85. Serrated teeth (D57):

0. absent
1. present

86. Teeth in premaxilla and cranial part of dentary (D9):

0. present
1. absent

87. Premaxillary teeth, numbers on each side (B15):

Protorosaurus is recoded from ? to 0 based on Gottmann-Quesada and Sander[8]. *Dinocephalosaurus* is recoded from 0 to ?.

0. seven or fewer
1. more than seven

88. Maxillary tooth plate (D60):

Jesairosaurus is recoded from ? to 0 based on the personal observation of MNHN ZAR 07.

0. absent
1. present

89. Maxillary tooth rows (D61):

0. single
1. multiple

90. Maxillary grooves (D62):

Dinocephalosaurus is recoded from ? to 0.

0. none
1. one
2. two

91. Maxillary teeth location (D63):

0. only on occlusal surface

1. on occlusal and lingual surfaces

92. Dentary tooth rows (D64):

0. one
1. two
2. more than two

93. Pterygoid teeth (Modified after B16, D68, D69):

Jesairosaurus is recoded from ? to 0 based on the personal observation of MNHN ZAR 07.

0. present
1. absent

94. Parasphenoid teeth (D44):

0. present
1. absent

95. Vomerine teeth (D66):

Dinocephalosaurus is recoded from 0 to 1.

0. present
1. absent

96. Palatine teeth (D67):

Tanystropheus is recoded from 1 to 0 based on Nosotti[14]. *Dinocephalosaurus* is recoded from 0 to 1.

0. present
1. absent

97. Vertebrae in adult (J29, D83):

0. notochordal
1. non-notochordal

98. Intervertebral articulation formed by zygosphene-zygantrum (J20):

0. no
1. yes

99. Neurocentral suture (D131):

Jesairosaurus and *Dinocephalosaurus* are recoded from ? to 0.

0. closed in adult
1. open in adult

100. Neural spine, ovoid spine-table on top (B21):

0. absent
1. present

101. Cervical vertebrae numbers (B17):

0. seven or fewer
1. more than seven

102. Cervical vertebra numbers (B18):

0. fewer than ten
1. ten or more

103. Thirteen cervical vertebrae:

0. absent
1. present

104. Atlas pleurocentrum, odontoid prominence (D133):

0. absent
1. present

105. Postaxial cervical intercentra (D79):

Prolacerta is recoded from 1 to 0 following Modesto and Sues[7].

0. present
1. absent

106. Cervical neural spine shape (B20, D28):

0. short and tall
1. long and low

107. Cervical neural spines, distal ends (D143):

0. no expansion
1. expansion present in form of flat table

108. Cervical neural arch, cranial margin (D134):

Dinocephalosaurus is recoded from ? to 1.

0. straight
1. notched to form overhang

109. Mid and posterior cervical and dorsal vertebral centra, relative lengths (B19, D81):

Jesairosaurus is recoded from ? to 0.

0. subequal
1. cervical centra longer

110. Dorsal neural spine height (Modified after B23, D85):

0. tall
1. subequal to length
2. low with height less than length

111. First five to six dorsal neural spines, distal ends (D140):

0. not expanded
1. expanded

112. Trunk vertebrae, transverse process well developed (J31):

Boreopricea is recoded from ? to 1. *Dinocephalosaurus* is recoded from 0 to 1.

0. no
1. yes

113. Trunk intercentra (B24, D80):

0. present
1. absent

114. Sacral vertebrae number:

0. two
1. three

115. First caudal (D128):

0. separate from sacrum
1. incorporated into sacrum

116. Caudal neural spines, distal ends (D139):

0. not expanded
1. expanded

117. Proximal caudal neural spine height (D88):
Petrolacosaurus is recoded from 1 to 0. *Youngina* is recoded from 0 to1. *Macrocnemus* is recoded from 0 to 1. *Langobardisaurus* is recoded from 0 to 1. *Tanystropheus* is recoded from 0 to 1. *Euparkeria* is recoded from 2 to 0. *Jesairosaurus* is recoded from 0 to 2.
0. moderately tall with height/length > 1and < 2
1. low with height/length < 1
2. tall with height/length > 2 and < 3
3. very tall with height/length > 3

118. Caudal transverse processes and centra, ratio of lengths (D89):
Youngina and *Rhynchosaurus* are recoded from 0 to 1.
0. equal or less than 1
1. more than 1

119. Caudal zygapophysis, articular facets (Modified after D129):
Langobardisaurus is recoded from ? to 0 based on the personal observation of MFSN 1921.
0. inclined
1. nearly or fully vertical

120. Haemal spine, distal width (D91):
Langobardisaurus is recoded from ? to 1+2.
0. equivalent to proximal width
1. tapering
2. wider than proximal width

121. Haemal spines, curvature (D141):
0. no curvature
1. cranial curvature present

122. Cervical ribs:
0. present
1. absent

123. Atlantal ribs:
0. present
1. absent

124. Axial ribs:
0. present
1. absent

125. Cervical ribs (B22, D77):
Euparkeria is recoded from ? to 1.
0. short and stout
1. long and slender

126. Tapering cervical ribs oriented posteriorly parallel to neck axis (J36, D77):
Trilophosaurus is recoded from 1 to 0. *Proterosuchus* is recoded from 0 to 1.

0. no
1. yes

127. Most cervical ribs dichocephalous (Modified after J30):

0. no
1. yes

128. Cervical ribs with anterior process (J37, D78):

0. no
1. yes

129. Anterior dorsal ribs (Modified after J19, D86):

Jesairosaurus is recoded from ? to 0 based on personal observation of MNHN ZAR 06.

0. dichocephalous
1. holocephalous

130. Posterior dorsal ribs (Modified after J19, D86):

0. dichocephalous
1. holocephalous

131. Posterior dorsal vertebrae and ribs (B25, D137):

Trilophosaurus is recoded from 0 to 1. *Dinocephalosaurus* is recoded from ? to 0.

0. not fused
1. fused

132. Second sacral rib, distal end (Modified after D87):

Langobardisaurus is recoded from 1 to 0+1.

0. not bifurcate
1. bifurcate

133. Sacral and caudal ribs (D132):

0. fused to centra
1. free

134. Proximal caudal ribs/transverse processes (Modified after D90):

0. recurved
1. project laterally

135. Gastralia (D92):

Prolacerta is temporarily recoded as ? here since gastralia have never been reported in a *Prolacerta* specimen.

0. absent
1. present

136. Cleithrum (J9, D93):

0. present
1. absent

137. Scapular blade shape (D99, B26, J45):

Dinocephalosaurus is recoded as 1 based on an unpublished specimen currently in a private collection.

0. tall and rectangular
1. low

2. tall and very narrow

138. Scapula and coracoid in adult:

0. fused
1. separate

139. Clavicular shape (D95):

Langobardisaurus is recoded as ?.

0. broad proximally
1. narrow proximally

140. Interclavicle, cranial margin (D97):

Langobardisaurus is recoded from ? to 0 based on MCSNB 2883.

0. smoothly convex
1. notch present between clavicles

141. Interclavicle, caudal stem (D98):

Euparkeria is recoded as 1.

0. little change in width along entire length
1. expansion present

142. Entepicondylar foramen (B27, D107):

0. present
1. absent

143. Ectepicondylar foramen (J21, D108):

Boreopricea is coded as 0.

0. absent
1. present

144. Radius length relative to humerus (B28):

Trilophosaurus is recoded from 1 to 0.

0. radius 80-90 percent length of humerus
1. radius 40-65 percent length of humerus

145. Intermedium in carpus (B29, J47):

0. present
1. absent

146. Centralia in the manus (B30, J10, D109):

0. present
1. absent

147. First distal carpal (B31):

Rhynchosaurus is recoded from 1 to 0. *Megalancosaurus* is recoded from ? to 0.

0. present
1. absent

148. Metacarpals 3 and 4, relative lengths (B32):

Protorosaurus is recoded from ? to 1.

0. metacarpal 3 shorter than 4
1. metacarpal 3 equal in length to, or longer than, 4

149. Metacarpals 1 and 5, relative lengths (B33):

Protorosaurus and *Tanytrachelos* are recoded from 1 to 0.

0. shorter than metacarpals 2 and 4
1. similar in length to metacarpals 2 and 4

150. Pelvic girdle (B36, D100):
0. unfenestrated
1. thyroid fenestra present

151. Acetabulum (D101):
0. elongate
1. circular

152. Acetabulum, relative contributions of pubic elements (J48, D105):
0. primarily the ilium
1. approximately equal contributions from each element

153. Ilium, dorsal margin (D102, B35):
Langobardisaurus is recoded from ? to 1.
0. posterior process only
1. large posterior process and smaller anterior process
2. equally developed anterior and posterior processes
3. large anterior projection

154. Ilium, pubic apron (D144):
0. absent
1. present

155. Pubis shape (B37):
Megalancosaurus is recoded from 0 to 1. *Jesairosaurus* is recoded from 0 to ?.
0. broad
1. narrow and waisted

156. Pubis, processus lateralis (D103):
0. present
1. absent

157. Pubis, anterior apron (D104):
0. absent
1. present

158. Femoral humeral ratio of lengths (D106):
Jesairosaurus is recoded from 0 to 1.
0. 1:1
1. femur > humerus

159. Femur shape (B38):
Rhynchosaurus and *Dinocephalosaurus* are recoded from 1 to 0.
Langobardisaurus is recoded from 0 to 1. *Jesairosaurus* is recoded from 1 to ?.
Boreopricea is recoded from 0 to ?.
0. sigmoidal
1. straight

160. Femoral distal surfaces (D110):
Tanystropheus is recoded from 0 to 1. *Boreopricea* is recoded from 1 to ?.
0. unequal in size
1. equal in size

161. **Femur, relative proportions (D111):**

0. distal width/total length equal or less than 0.3
1. distal width/total length more than 0.3

162. **Tibia and femur, relative lengths (B39):**

Tanytrachelos is recoded from 1 to 0.

0. tibia shorter than, or subequal to, femur in length
1. tibia longer

163. **Ossified tarsals in adult (Modified after J52):**

0. five or more
1. less than five

164. **Ankle, foramen between astragalus and calcaneum (B40, D115):**

0. present
1. absent

165. **Concavo-convex astragalo-calcaneal articulation (J34, D113):**

0. absent
1. present

166. **Astragalus and calcaneum (J23):**

0. unfused
1. fused

167. **Astragalus shape (Modified after D135):**

Boreopricea is recoded from ? to 0.

0. L-shaped with broad base
1. elongate
2. round

168. **Lateral calcaneal tuber (B41, D116):**

Mesosuchus is recoded from 0 to 1.

0. absent
1. present

169. **"Lepidosauromorph" ankle joint where fourth distal tarsal has dorsal process fitting into recess on astragalo-calcaneum (J24, D114):**

0. no
1. yes

170. **Pes centrale (B42):**

Proterosuchus is recoded from 1 to 0.

0. present
1. absent

171. **Pes centrale (D117):**

Trilophosaurus is recoded from 1 to 0.

0. present and does not contact tibia
1. present and contacts tibia

172. **Pes centrale (D118):**

Youngina and *Proterosuchus* are recoded from 0 to 1. *Mesosuchus* is recoded from 1 to 0+1.

0. present and contacts distal tarsal 4

1. present and does not contact distal tarsal 4

173. First distal tarsal (B43, D119):

Langobardisaurus is recoded from ? to 1.

0. present
1. absent

174. Second distal tarsal (B44, D120) :

0. present
1. absent

175. Fifth distal tarsal (J11, D121):

Youngina and *Mesosuchus* are recoded from 0 to 1.

0. present
1. absent

176. Metatarsals I and IV, ratio of lengths (D123):

0. equal or more than 0.4
1. equal or more than 0.3 but less than 0.4
2. less than 0.3

177. Metatarsals 4 and 5, relative lengths (B45):

Proterosuchus and *Protorosaurus* are recoded from 1 to 0.

0. metatarsal 4 less than three times length of metatarsal 5
1. metatarsal 4 more than three times length of metatarsal 5

178. Fifth metatarsal (D122, B46):

0. straight
1. hooked without deflection
2. hooked with deflection of proximal head

179. Digits 3 and 4 of foot, ratio of lengths (D124):

Langobardisaurus is recoded from 1 to 2.

0. equal or less than 0.8
1. more than 0.8 but less than 0.9
2. equal or more than 0.9

180. Digit 5, first phalanx (D138, J71):

0. shorter than or equal to length of first metatarsal
1. significantly longer than the first metatarsal

181. Hyperphalangy in pes:

0. absent
1. present

182. Postcloacal bones (B48):

0. absent
1. present

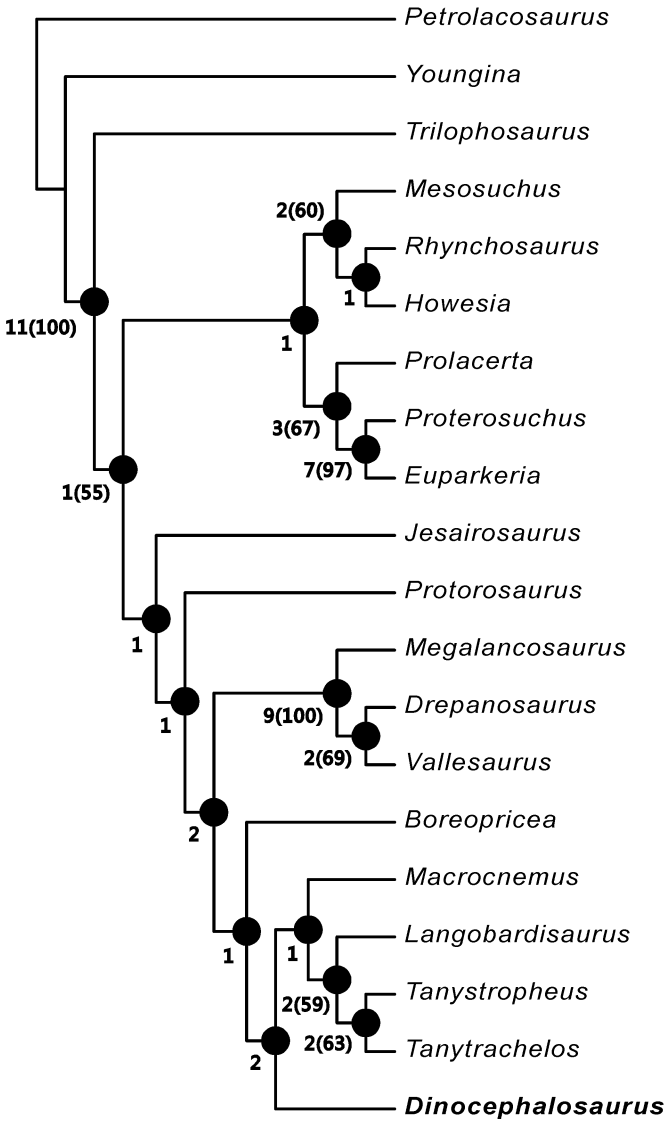

Supplementary Figure 1 | Phylogenetic hypothesis of *Dinocephalosaurus* inferred by parsimony. The number inside the bracket is the bootstrap value (1000 replications of Branch and Bound search with other settings as default). Only bootstrap proportions greater than 50% are indicated, while the number beside the bracket is the decay index (Bremer support).

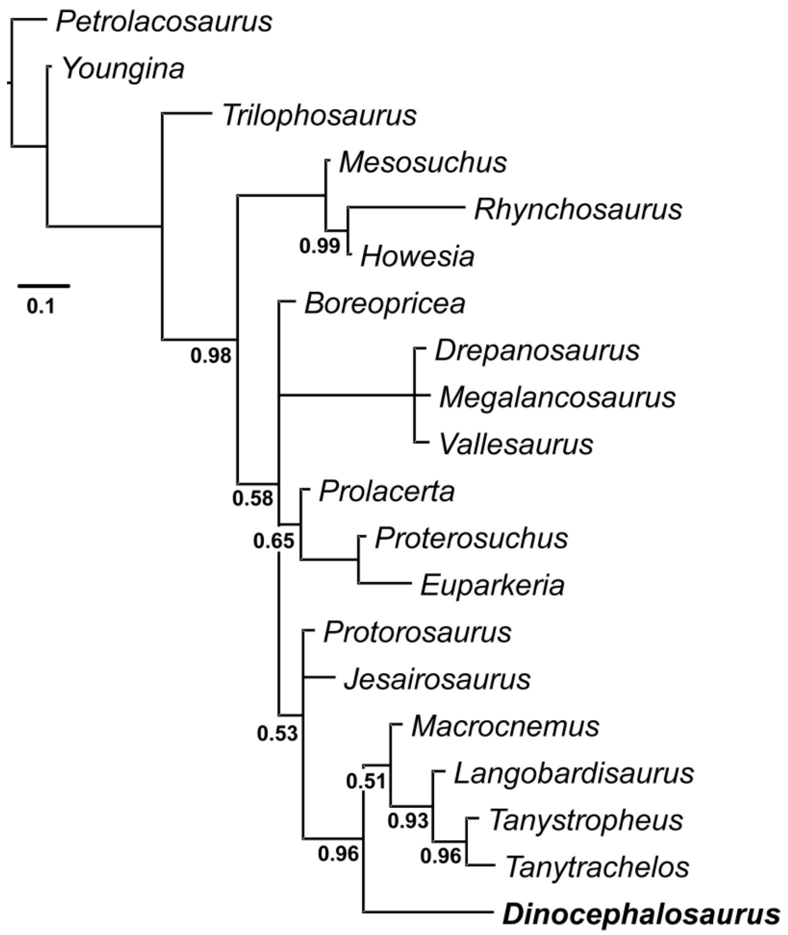

Supplementary Figure 2 | Phylogenetic hypothesis of *Dinocephalosaurus* inferred by Bayesian analysis. Nodes without labels are supported with a posterior probability of 1.0. Multifurcations without labels have posterior support of less than 0.5. Otherwise nodes are labelled according to their posterior probability. Short terminal branch lengths are the result of poorly sampled autapomorphies.

Supplementary Table 1 | List of specimens examined personally by J.L. and literature referred to. Institutional abbreviations: FMNH, Field Museum of Natural History, Chicago, USA; GMPKU, Geological Museum of Peking University, Beijing, China; IVPP, Institute of Vertebrate Palaeontology and Palaeoanthropology, Beijing, China; CCCGS, Chengdu Center of China Geological Survey, Chengdu, China; MCSNB, Museo Civico di Scienze Naturale Enrico Caffi, Bergamo, Italy; MFSN, Museo Friulano di Storia Naturale, Udine, Italy; MNHN, Muséum National d'Histoire Naturelle, Paris, France; MSNM, Museo di Storia Naturale di Milano, Milan, Italy; NHMW, Naturhistorisches Museum Wien, Vienna, Austria; PIMUT, Paleontological Institute and Museum, University of Zurich, Zurich, Switzerland.

Taxon	Specimens	Relevant literature
Petrolacosaurus		(Reisz, 1981)[15]
Youngina		(Gow, 1975)[6];<br>(Carroll, 1981)[10];<br>(Currie, 1981)[16];<br>(Smith and Evans, 1996)[17]
Trilophosaurus	FMNH PR259 (cast)	(Gregory, 1945)[9];<br>(Demar and Bolt, 1981)[18];<br>(Murry, 1987)[19];<br>(Heckert *et al*., 2001)[20];<br>(Spielmann *et al*., 2005)[21];<br>(Heckert *et al*., 2006)[22];<br>(Mueller and Parker, 2006)[23];<br>(Spielmann *et al*., 2007)[24]<br>(Nesbitt *et al*., 2015)[25]
Rhynchosaurus		(Benton, 1990)[5]
Mesosuchus		(Dilkes, 1998)[3]
Howesia		(Dilkes, 1995)[12]
Drepanosaurus	MCSNB 5728	(Pinna, 1986)[26];<br>(Renesto, 1994)[27];<br>(Renesto and Paganoni, 1995)[28];<br>(Pritchard *et al*., 2016)[29]
Megalancosaurus	MFSN 1769;<br>MFSN 1801;<br>MFSN 184432	(Calzavara *et al*., 1980)[30];<br>(Feduccia and Wild, 1993)[31];<br>(Renesto, 1994)[32];<br>(Renesto, 2000)[33];<br>(Renesto and Dalla Vecchia, 2005)[13]
Vallesaurus	MCSNB 4751	(Renesto and Binelli, 2006)[34]

Taxon	Specimens	Relevant literature
Macrocnemus	PIMUT Z 1559; PIMUT Z 2470; PIMUT Z 2472; PIMUT Z 2473; PIMUT Z 2474; PIMUT Z 2475; PIMUT Z 2476; PIMUT Z 2816; PIMUT Z 5918; PIMUZ T 2477; PIMUZ T 4822; GMPKU-P-3001; IVPP V 15001	(Peyer, 1937)[35]; (Kuhn-Schnyder, 1962)[36]; (Rieppel and Gronowske, 1981)[37]; (Rieppel, 1989)[38]; (Li *et al*., 2007)[39]; (Zhang *et al*., 2010)[40]; (Jiang *et al*., 2011)[41]; (Fraser and Furrer, 2013)[42]; (Pritchard *et al*., 2015)[43]
Langobardisaurus	MCSNB 2883; MCSNB 4860; MFSN 1921	(Renesto, 1994)[44]; (Renesto and Dalla Vecchia, 2000)[45]; (Renesto *et al*., 2002)[46]; (Saller *et al*., 2013)[47]; (Pritchard *et al*., 2015)[43]
Tanystropheus	MSNM BES SC 111; MSNM BES SC 265; MSNM BES SC 1018; MSNM V3663; MSNM V3730; MFSN 25761; MFSN 26829; MFSN 31573; PIMUT Z 2482; PIMUT Z 2483; PIMUT Z 2484; PIMUT Z 2790; PIMUZ T 1277; PIMUZ T 2791; GMPKU-P-1527; IVPP V 14472	(Peyer, 1931)[48]; (Wild, 1973, 1980a, 1980b)[49, 50, 51]; (Wild and Oosterink, 1984)[52]; (Dalla Vecchia, 2000)[53]; (Rieppel, 2001)[54]; (Renesto, 2005)[55]; (Dalla Vecchia, 2006)[56]; (Li, 2007)[57]; (Nosotti, 2007)[14]; (Rieppel *et al*., 2010)[58]; (Pritchard *et al*., 2015)[43]
Tanytrachelos		(Olsen, 1979)[59]; (Casey *et al*., 2007)[60]; (Pritchard *et al*., 2015)[43]
Dinocephalosaurus	IVPP V13767; IVPP V13898; CCCGS LPV 30280; CCCGS LPV 30174	(Li, 2003)[61]; (Li et al., 2004)[62]; (Rieppel *et al*., 2008)[4]
Boreopricea		(Tatarinov, 1978)[63]; (Benton and Allen, 1997)[1]

Taxon	Specimens	Relevant literature
Protorosaurus	NHMW 1943I4;<br>NHMW 1974-1635 (cast)	(Seeley, 1887)[64];<br>(Romer, 1947)[65];<br>(Evans and King, 1993)[66];<br>(Gottmann -Quesada and Sander, 2009)[8]
Jesairosaurus	MNHN ZAR 6;<br>MNHN ZAR 7;<br>MNHN ZAR 8;<br>MNHN ZAR 9;<br>MNHN ZAR 10;<br>MNHN ZAR 11;<br>MNHN ZAR 12;<br>MNHN ZAR 13;<br>MNHN ZAR 14;<br>MNHN ZAR 15	(Jalil, 1997)[2]
Prolacerta		(Parrington, 1935)[67];<br>(Camp, 1945a, 1945b)[68, 69];<br>(Gow, 1975)[6];<br>(Evans, 1986)[70];<br>(Colbert, 1987)[71];<br>(Modesto and Sues, 2004)[7];<br>(Botha -Brink and Smith, 2011)[72]
Proterosuchus		(Cruickshank, 1972)[73];<br>(Clark et al., 1993)[74];<br>(Welman, 1998)[75];<br>(Klembara and Welman, 2009)[76];<br>(Botha-Brink and Smith, 2011)[72]
Euparkeria		(Ewer, 1965)[11];<br>(Clark *et al*., 1993)[74];<br>(Gower and Weber, 1998)[77];<br>(Senter, 2003)[78];<br>(Botha-Brink and Smith, 2011)[72]

Supplementary References

1. Benton MJ, Allen JL. *Boreopricea* from the Lower Triassic of Russia, and the relationships of the prolacertiform reptiles. *Palaeontology* **40**, 931-953 (1997).

2. Jalil NE. A new prolacertiform diapsid from the Triassic of North Africa and the interrelationships of the Prolacertiformes. *J. Vertebr. Paleontol.* **17**, 506-525 (1997).

3. Dilkes DW. The Early Triassic rhynchosaur *Mesosuchus browni* and the interrelationships of basal archosauromorph reptiles. *Philos. T. Roy. Soc. B* **353**, 501-541 (1998).

4. Rieppel O, Li C, Fraser NC. The skeletal anatomy of the Triassic protorosaur *Dinocephalosaurus orientalis* Li, from the Middle Triassic of Guizhou Province, southern China. *J. Vertebr. Paleontol.* **28**, 95-110 (2008).

5. Benton MJ. The species of *Rhynchosaurus*, a rhynchosaur (Reptilia, Diapsida) from the Middle Triassic of England. *Philos. T. Roy. Soc. B* **328**, 213-306 (1990).

6. Gow C. The morphology and relationships of *Youngina capensis* Broom and *Prolacerta broomi* Parrington. *Palaeontologia Africana* **18**, 89-131 (1975).

7. Modesto SP, Sues H-D. The skull of the Early Triassic archosauromorph reptile *Prolacerta broomi* and its phylogenetic significance. *Zool. J. Linn. Soc.* **140**, 335-351 (2004).

8. Gottmann-Quesada A, Sander PM. A redescription of the early archosauromorph *Protorosaurus speneri* Meyer,1832,and its phylogenetic relationships. *Palaeontogr. Abt. A* **287**, 123-220 (2009).

9. Gregory JT. Osteology and relationships of *Trilophosaurus*. *University of Texas Publication* **4401**, 273-359 (1945).

10. Carroll RL. Plesiosaur ancestors from the Upper Permian of Madagascar. *Philos T Roy Soc B* **293**, 315-383 (1981).

11. Ewer RF. The anatomy of the thecodont reptile *Euparkeria capensis* Broom. *Philos. T. Roy. Soc. B* **248**, 379-435 (1965).

12. Dilkes DW. The rhynchosaur *Howesia browni* from the Lower Triassic of South Africa. *Palaeontology* **38**, 665-685 (1995).

13. Renesto S, Dalla Vecchia FM. The skull and lower jaw of the holotype of

Megalancosaurus preonensis (Diapsida, Drepanosauridae) from the Upper Triassic of Northern Italy. *Riv. Ital. Paleontol. S.* **111**, 247-257 (2005).

14. Nosotti S. *Tanystropheus longobardicus* (Reptilia, Protorosauria): re-interpretations of the anatomy based on new specimens from the Middle Triassic of Besano (Lombardy, Northern Italy). *Memorie della Società Italiana di Scienze Naturali e del Museo Civico di Storia Naturale di Milano Memorie* **35**, 1-88 (2007).

15. Reisz R. A diapsid reptile from the Pennsylvanian of Kansas. *Special Publication of the Museum of Natural History, University of Kansas* **7**, 1-74 (1981).

16. Currie PJ. The vertebrae of *Youngina* (Reptilia: Eosuchia). *Can. J. Earth Sci.* **18**, 815-818 (1981).

17. Smith RMH, Evans SE. New material of *Youngina*: evidence of juvenile aggregation in Permian diapsid reptiles. *Palaeontology* **39**, 289-304 (1996).

18. Demar R, Bolt JR. Dentitional organization and function in a Triassic reptile. *J. Paleontol.* **55**, 967-984 (1981).

19. Murry PA. New reptiles from the Upper Triassic Chinle Formation of Arizona. *J. Paleontol.* **61**, 773-786 (1987).

20. Heckert A, Lucas S, Kahle R, Zeigler K. New occurrence of *Trilophosaurus* (Repitilia: Archosauromorpha) from the Upper Triassic of West Texas and its biochronological significance. *Geology of the Llano Estacado: New Mexico Geological Society Guidebook* **52**, 115-122 (2001).

21. Spielmann JA, Heckert AB, Lucas SG. The Late Triassic archosauromorph *Trilophosaurus* as an arboreal climber. *Riv. Ital. Paleontol. S.* **111**, 395-412 (2005).

22. Heckert AB, Lucas SG, Rinehart LF, Spielmann JA, Hunt AP, Kahle R. Revision of the archosauromorph reptile *Trilophosaurus*, with a description of the first skull of *Trilophosaurus jacobsi*, from the Upper Triassic Chinle Group, West Texas, USA. *Palaeontology* **49**, 621-640 (2006).

23. Mueller BD, Parker WG. A new species of *Trilophosaurus* (Diapsida: Archosauromorpha) from the Sonsela Member (Chinle Formation) of Petrified Forest National Park, Arizona. *Museum of Northern Arizona, Bulletin* **62**, 119-125 (2006).

24. Spielmann JA, Lucas SG, Heckert AB, Rinehart LF, Hunt A. Taxonomy and biostratigraphy of the Late Triassic archosauromorph *Trilophosaurus*. *New Mexico Museum of Natural History and Science Bulletin* **40**, 231-240 (2007).

25. Nesbitt SJ, Flynn JJ, Pritchard AC, Parrish JM, Ranivoharimanana L, Wyss AR. Postcranial osteology of *Azendohsaurus madagaskarensis* (?Middle to Upper Triassic, Isalo Group, Madagascar) and its systematic position among stem archosaur reptiles. *B Am. Mus. Nat. Hist.* **398**, 1-126 (2015).

26. Pinna G. On *Drepanosaurus unguicaudatus*, an Upper Triassic lepidosaurian from the Italian Alps. *J. Paleontol.* **60**, 1127-1132 (1986).

27. Renesto S. The shoulder girdle and anterior limb of *Drepanosaurus unguicaudatus* (Reptilia, Neodiapsida) from the upper Triassic (Norian) of Northern Italy. *Zool. J. Linn. Soc.* **111**, 247-264 (1994).

28. Renesto S, Paganoni A. A new *Drepanosaurus* (Reptilia, Neodiapsida) from the Upper Triassic of Northern Italy. *Neues Jahrbuch für Geologie und Paleontologie, Abhandlungen* **197**, 87-100 (1995).

29. Pritchard Adam C, Turner Alan H, Irmis Randall B, Nesbitt Sterling J, Smith Nathan D. Extreme modification of the tetrapod forelimb in a Triassic diapsid reptile. *Curr. Biol.* **26**, 2779-2786 (2016).

30. Calzavara M, Muscio G, Wild R. *Megalancosaurus preonensis* n.g., n. sp., a new reptile from the Norian of Friuli. *Gortania* **2**, 49-64 (1980).

31. Feduccia A, Wild R. Birdlike characters in the Triassic archosaur *Megalancosaurus*. *Naturwissenschaften* **80**, 564-566 (1993).

32. Renesto S. *Megalancosaurus*, a possibly arboreal archosauromorph (Reptilia) from the Upper Triassic of Northern Italy. *J. Vertebr. Paleontol.* **14**, 38-52 (1994).

33. Renesto S. Bird-like head on a chameleon body: new specimens of the enigmatic diapsid reptile *Megalancosaurus* from the Late Triassic of Northern Italy. *Riv. Ital. Paleontol. S.* **106**, 157-180 (2000).

34. Renesto S, Binelli G. *Vallesaurus cenensis* Wild, 1991, a drepanosaurid (Reptilia, Diapsida) from the Late Triassic of northern Italy. *Riv. Ital. Paleontol. S.* **112**, 77-94 (2006).

35. Peyer B. Die Triasfauna der Tessiner Kalkalpen. XII. *Macrocnemus bassanii* Nopcsa. *Abhandlungen der schweizerischen paläontologischen Gesellschaft*

59, 1-140 (1937).

36. Kuhn-Schnyder E. Ein weiterer Schädel von *Macrocnemus bassanii* Nopcsa aus der anisischen Stufe der Trias des Monte San Giorgio (Kt. Tessin, Schweiz). *Paläontologische Zeitschrift* **36**, 110-133 (1962).

37. Rieppel O, Gronowske RW. The loss of the lower temporal arcade in diapsid reptiles. *Zool. J. Linn. Soc.* **72**, 203-217 (1981).

38. Rieppel O. The hind limd of *Macronemus bassanii* (Nopcsa) (Reptilia, Diapsida): deverlopment and functional anatomy. *J. Vertebr. Paleontol.* **9**, 373-387 (1989).

39. Li C, Zhao LJ, Wang LT. A new species of *Macrocnemus* (Reptilia: Protorosauria) from the Middle Triassic of southwestern China and its palaeogeographical implication. *Sci. China Ser. D* **50**, 1601-1605 (2007).

40. Zhang BM, Chen XH, Cheng L. Discovery of *Macrocnemus* cf. *fuyuanensis* of the Middle Triassic in Xinyi, Guizhou Province, SW China. *Geology and Mineral Resources of South China* **2010**, 43-47 (2010).

41. Jiang DY, *et al.* New information on the protorosaurian reptile *Macrocnemus fuyuanensis* Li et al., 2007, from the Middle/Upper Triassic of Yunnan, China. *J. Vertebr. Paleontol.* **31**, 1230-1237 (2011).

42. Fraser N, Furrer H. A new species of *Macrocnemus* from the Middle Triassic of the eastern Swiss Alps. *Swiss J. Geosci.* **106**, 199-206 (2013).

43. Pritchard AC, Turner AH, Nesbitt SJ, Irmis RB, Smith ND. Late Triassic tanystropheids (Reptilia, Archosauromorpha) from northern New Mexico (Petrified Forest Member, Chinle Formation) and the biogeography, functional morphology, and evolution of Tanystropheidae. *J. Vertebr. Paleontol.* **35**, e911186 (2015).

44. Renesto S. A new prolacertiform reptile from the Late Triassic of Northern Italy. *Riv. Ital. Paleontol. S.* **100**, 285-306 (1994).

45. Renesto S, Dalla Vecchia FM. The unusual dentition and feeding habits of the prolacertiform reptile *Langobardisaurus* (Late Triassic, Northern Italy). *J. Vertebr. Paleontol.* **20**, 622-627 (2000).

46. Renesto S, Dalla Vecchia FM, Peters D. Morphological evidence for bipedalism in the Late Triassic prolacertiform reptile *Langobardisaurus*. *Senckembergiana Lethaea* **82**, 95-106 (2002).

47. Saller F, Renesto S, Dalla Vecchia FM. First record of *Langobardisaurus* (Diapsida, Protorosauria) from the Norian (Late Triassic) of Austria, and a revision of the genus. *Neues Jahrbuch für Geologie und Paläontologie, Abhandlungen* **268**, 83-95 (2013).

48. Peyer B. Die Triasfauna der Tessiner Kalkalpen. II. *Tanystropheus longobardicus* Bass. sp. *Abhandlungen der schweizerischen paläontologischen Gesellschaft* **50**, 6-110 (1931).

49. Wild R. Die Triasfauna der Tessiner Kalkalpen. XXIII. *Tanystropheus longobardicus* (Bassani) (Neue Ergebnisse). *Schweizerische Paläontologische Abhandlungen* **95**, 1-162 (1973).

50. Wild R. Die Triasfauna der Tessiner Kalkalpen XXIV. Neue Funde von *Tanystropheus* (Reptilia, Squamata). *Schweizerische Paläontologische Abhandlungen* **102**, 1-43 (1980).

51. Wild R. *Tanystropheus* (Reptilia: Squamata) and its importance for stratigraphy. *Mémoires de la Société Géologique de France NS* **139**, 201-206 (1980).

52. Wild R, Oosterink H. *Tanystropheus* (Reptilia, Squamata) aus dem Unteren Muschelkalk von Winterswijk, Holland. *Grondboor en Hamer* **5**, 142-148 (1984).

53. Dalla Vecchia FM. *Tanystropheus* (Archosauromorpha, Prolacertiformes) remains from the Triassic of the Northern Friuli (NE Italy). *Riv. Ital. Paleontol. S.* **106**, 135-140 (2000).

54. Rieppel O. A new species of *Tanystropheus* (Reptilia: Protorosauria) from the Middle Triassic of Makhtesh Ramon, Israel. *Neues Jahrbuch für Geologie und Paläontologie, Abhandlungen* **221**, 271-287 (2001).

55. Renesto S. A new specimen of *Tanystropheus* (Reptilia Protorosauria) from the Middle Triassic of Switzerland and the ecology of the genus. *Riv. Ital. Paleontol. S.* **111**, 377-394 (2005).

56. Dalla Vecchia FM. Resti di *Tanystropheus*, saurotterigi e “rauisuchi” (Reptilia) nel Triassico medio della Val Aupa (Moggio Udinese, Udine). *Gortania. Atti. Mus. Friul. St. Nat.* **27**, 25-48 (2006).

57. Li C. A juvenile *Tanystropheus* sp. (Protorosauria, Tanystropheidae) from the Middle Triassic of Guizhou, China. *Vertebrata PalAsiatica* **45**, 37-42 (2007).

58. Rieppel O, *et al. Tanystropheus* cf. *T. longobardicus* from the early Late Triassic of Guizhou Province, southwestern China. *J. Vertebr. Paleontol.* **30**, 1082-1089 (2010).

59. Olsen PE. A new aquatic eosuchian from the Newark supergroup (Late Triassic-Early Jurassic) of North Carolina and Virginia. *Postilla* **176**, 1-14 (1979).

60. Casey MM, Fraser NC, Kowalewski M. Quantitative taphonomy of a Triassic reptile *Tanytrachelos ahynis* from the Cow Branch Formation, Dan River Basin, Solite Quarry, Virginia. *Palaios* **22**, 598 (2007).

61. Li C. First record of protorosaurid reptile (Order Protorosauria) from the Middle Triassic of China. *Acta Geol. Sin.-Engl.* **77**, 419-423 (2003).

62. Li C, Rieppel O, LaBarbera MC. A Triassic aquatic protorosaur with an extremely long neck. *Science* **305**, 1931-1931 (2004).

63. Tatarinov L. Triassic prolacertilians of the USSR. *Paleontol. J.* **12**, 505-514 (1978).

64. Seeley HG. Researches on the structure, organization, and classification of the fossil Reptilia. I. On *Protorosaurus speneri* (von Meyer). *Philos. T. Roy. Soc. B* **178**, 187-213 (1887).

65. Romer AS. The relationships of the Permian reptile *Protorosaurus*. *Am. J. Sci.* **245**, 19-30 (1947).

66. Evans SE, King MS. A new specimen of *Protorosaurus* (Reptilia: Diapsida) from the Marl Slate (late Permian) of Britain. *Proceedings of the Yorkshire Geological and Polytechnic Society* **49**, 229-234 (1993).

67. Parrington FR. On *Prolacerta broomi* gen et sp. n., and the origin of lizards. *Annals and Magazine of Natural History (10)* **16**, 197-205 (1935).

68. Camp CL. *Prolacerta* and the protorosaurian reptiles. Part 1. *Am. J. Sci.* **243**, 17-32 (1945).

69. Camp CL. *Prolacerta* and the protorosaurian reptiles. Part 2. *Am. J. Sci.* **243**, 84-101 (1945).

70. Evans SE. The braincase of *Prolacerta broomi* (Reptilia: Triassic). *Neues Jahrbuch für Geologie und Paläontologie, Abhandlungen* **173**, 181-200

(1986).

71. Colbert EH. The Triassic reptile *Prolacerta* in Antarctica. *Am. Mus. Novit.* **2882**, 1-19 (1987).

72. Botha-Brink J, Smith RMH. Osteohistology of the Triassic archosauromorphs *Prolacerta*, *Proterosuchus*, *Euparkeria*, and *Erythrosuchus* from the Karoo Basin of South Africa. *J. Vertebr. Paleontol.* **31**, 1238-1254 (2011).

73. Cruickshank ARI. The proterosuchian thecodonts. In: *Studies in Vertebrate Evolution* (ed^(eds Joysey KA, Kemp TS). Oliver & Boyd (1972).

74. Clark JM, Welman J, Gauthier JA, Parrish JM. The laterosphenoid bone of early archosauriforms. *J. Vertebr. Paleontol.* **13**, 48-57 (1993).

75. Welman J. The taxonomy of the South African proterosuchids (Reptilia, Archosauromorpha). *J. Vertebr. Paleontol.* **18**, 340-347 (1998).

76. Klembara J, Welman J. The anatomy of the palatoquadrate in the Lower Triassic *Proterosuchus fergusi* (Reptilia, Archosauromorpha) and its morphological transformation within the archosauriform clade. *Acta Zool.* **90**, 275-284 (2009).

77. Gower DJ, Weber E. The braincase of *Euparkeria*, and the evolutionary relationships of birds and crocodilians. *Biol. Rev.* **73**, 367-411 (1998).

78. Senter P. New information on cranial and dental features of the Triassic archosauriform reptile *Euparkeria capensis*. *Palaeontology* **46**, 613-621 (2003).
